# Dynamics of H3K27me3 Modification on Plant Adaptation to Environmental Cues

**DOI:** 10.3390/plants10061165

**Published:** 2021-06-08

**Authors:** Qingwen Shen, Yisheng Lin, Yingbo Li, Guifeng Wang

**Affiliations:** National Key Laboratory of Wheat and Maize Crops Science, CIMMYT-China (Henan) Joint Research Center of Wheat and Maize, Collaborative Innovation Center of Henan Grain Crops, College of Agronomy, Henan Agricultural University, Zhengzhou 450002, China; shenqingwen@henau.edu.cn (Q.S.); lysls18503801927@126.com (Y.L.); liybo2018@163.com (Y.L.)

**Keywords:** H3K27me3, epigenetics, environmental cues, polycomb repressive complex 2, polycomb repressive complex 1, demethylase, recruitment

## Abstract

Given their sessile nature, plants have evolved sophisticated regulatory networks to confer developmental plasticity for adaptation to fluctuating environments. Epigenetic codes, like tri-methylation of histone H3 on Lys27 (H3K27me3), are evidenced to account for this evolutionary benefit. Polycomb repressive complex 2 (PRC2) and PRC1 implement and maintain the H3K27me3-mediated gene repression in most eukaryotic cells. Plants take advantage of this epigenetic machinery to reprogram gene expression in development and environmental adaption. Recent studies have uncovered a number of new players involved in the establishment, erasure, and regulation of H3K27me3 mark in plants, particularly highlighting new roles in plants’ responses to environmental cues. Here, we review current knowledge on PRC2-H3K27me3 dynamics occurring during plant growth and development, including its writers, erasers, and readers, as well as targeting mechanisms, and summarize the emerging roles of H3K27me3 mark in plant adaptation to environmental stresses.

## 1. Introduction

Plants are sessile organisms that live tightly associated with a changing environment. To cope with the unfavorable stimuli, e.g., extreme temperature, osmotic stress, nutrient deficiency, and biotic stress, plants thus increase their phenotypic plasticity by extensive genetic and epigenetic reprograming in order to adapt to the environmental challenges [1,2]. Epigenetic codes mainly include DNA methylation, histone modifications, and small RNAs, which alter the structure and accessibility of chromatin, therefore either activating or inhibiting gene expression in a heritable mechanism [2,3].

DNA methylation at the carbon-5 position of cytosine (5mC) is a well-understood epigenetic mark that is relevant to gene repression and genome stability, involved in diverse biological processes, as well as in response to environmental cues [4,5]. Histone modifications, including methylation, acetylation, phosphorylation, ubiquitination, sumoylation, biotinylation, glycosylation, and ADP-ribosylation, are also conserved epigenetic marks in eukaryotes [6]. Dynamics of histone modifications are maintained by enzymes that are able to catalyze and remove the marks, referred to as writers and erasers, respectively. These epigenetic codes are in turn recognized by reader proteins and give rise to biological outcomes [3,7]. Further, several types of methylation of lysine residues of histone H3 are widespread and of great importance in plants, like repressive di-methylation of Lys9 (H3K9me2) and tri-methylation of Lys27 (H3K27me3), and permissive tri-methylation of Lys4 (H3K4me3) and tri-methylation of Lys36 (H3K36me3) [8].

An increasing number of recent studies have revealed that epigenetic mechanisms are also implicated in plants’ responses to environmental stresses; in particular, the roles of DNA methylation have been extensively discussed in several reviews [3,4]. By contrast, functions of H3K27me3 in plants’ adaption to environmental cues have not been well documented. In this review, we will discuss molecular machinery of H3K27me3 from de novo synthesis, removal, and genomic reorganization and loading in plants, emphasizing the potential regulatory importance of H3K27me3 dynamics on adaptation to various environmental cues.

## 2. Writers and Erasers of H3K27me3 in Plants

**Figure 1:**
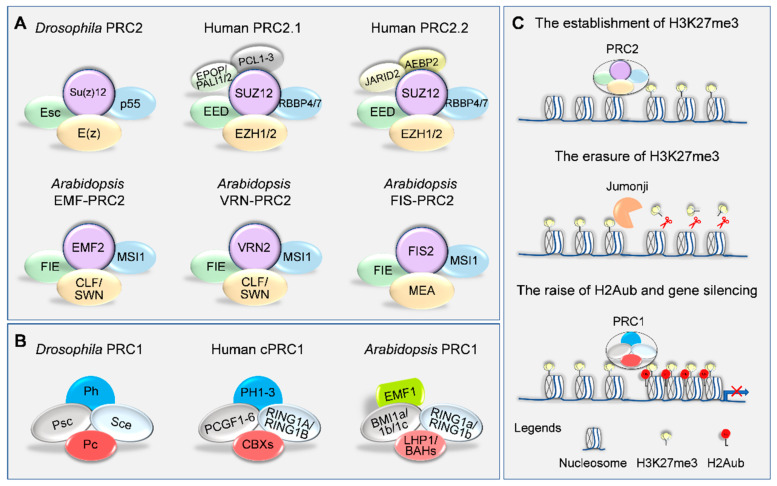
Core PRCs and their chromatin-modifying activities. (**A**,**B**) Compositions of the polycomb repressive complexes PRC2 (**A**) and PRC1 (**B**) in animals and plants, where Drosophila, human, and Arabidopsis were taken as representatives. (**C**) Schematic representation of the H3K27me3 deposition, erasure, and its roles in gene silencing. Acronyms and further details are explained in the text.

Basically, H3K27me3 is deposited on chromatin, covering thousands of genomic loci, including many developmental and stress-responsive genes in plants [9,10,11]. It is catalyzed by writer, polycomb repressive complex 2 (PRC2), via histone methyltransferases and related cofactors. PRC2 subunits were initially identified in *Drosophila melanogaster* and are well conserved across animal and plant kingdoms [12,13]. In *Drosophila*, PRC2 is composed of four core components, including the histone methyltransferase enhancer of zeste (E(z)), extra sex combs (Esc), suppressor of zeste 12 (Su(z)12), and the histone-binding nucleosome remodeling factor 55 kDa (Nurf55, also called p55) (Figure 1A) [14]. Specifically, E(z) belongs to the SET (Su(var)3-9; E(z); trithorax) domain family, responsible for tri-methylation on histone H3 at Lys27 [15], while Esc is a member of WD40 repeat family and possesses the β-propeller architecture that binds to the histone H3 tail [16]. Su(z)12 is a C2H2 zinc-finger protein with a VEFS (VRN2; EMF2; FIS2; Su(z)12) domain that interacts with E(z) and various cofactors [17]. Nurf55 is also a member of WD40 repeat-containing histone chaperone protein that can bridge PRC2 and chromatin together by directly binding to nucleosome, but is dispensable for PRC2-mediated gene silencing in vivo [18]. In comparison, mammalian core PRC2 subunits consist of enhancer of zeste homolog 2/1 (EZH2/1), embryonic ectoderm development (EED), suppressor of zeste 12 homolog (SUZ12), and retinoblastoma binding protein 4/7 (RBBP4/7) [19,20,21]. Associated with specific accessory proteins, two mutually exclusive subtype assemblies, PRC2.1 (PCL1–3, EPOP, and PALI1/2) and PRC2.2 (AEBP2 and JARID2), are formed and cooperate to mediate the deposition and maintenance of H3K27me3 via both synergistic and independent mechanisms (Figure 1A) [21,22,23].

Unlike animals, the core PRC2 components undergo multiple duplications in plants [24,25]. In the case of *Arabidopsis*, three E(z) homologs (CURLY LEAF (CLF), SWINGER (SWN), and MEDEA (MEA)), three Su(z)12 homologs (EMBRYONIC FLOWER 2 (EMF2), VERNALIZATION 2 (VRN2), and FERTILIZATION INDEPENDENT SEED 2 (FIS2)), one Esc homolog (FERTILIZATION-INDEPENDENT ENDOSPERM (FIE)), and five Nurf55 homologs (MULTICOPY SUPRESSOR OF IRA 1–5 (MSI1–5)) are present [26,27]. Based on cell or tissue specificity, three distinct PRC2s are formed, referred to as the EMF-, VRN-, and FIS-PRC2 complexes (Figure 1A). The FIS-PRC2, harboring FIS2, MEA, FIE, and MSI1, is implicated in regulating megagametogenesis and endosperm development postfertilization [28,29,30,31,32,33]. Whereas the EMF-PRC2 (having EMF2, CLF/SWN, FIE, and MSI1) and VRN-PRC2 (including VRN2, CLF/SWN, FIE, and MSI1) control sporophyte development and the vegetative-to-reproductive phase transition [33,34,35,36,37,38,39].

In cereals, rice (*Oryza sativa* L.) and maize (*Zea mays* L.) both have two copies of EMF2-like genes (EMF2a and EMF2b) but no VRN2 or FIS2 homologs [40]. For E(z), two (OsCLF and OsiEZ1) and three (ZmMEZ1–3) homologs are present in rice and maize, respectively [40]. Intriguingly, a duplicated pair of Esc homologs are found in rice (OsFIE1 and OsFIE2) and maize (ZmFIE1 and ZmFIE2), in contrast to a single FIE in *Arabidopsis* [24,40]. At present, three and seven MSI homologs have been identified from rice and maize, respectively [24,25]. This scenario suggests gene duplication is a major evolutionary force shaping plant PRC2, most likely in a species- or genus-specific manner (Table 1). Nevertheless, the distinct PRC2 complexes and their functions in cereal growth and development remain to be elucidated [24,25,40,41,42,43,44].

To maintain H3K27me3 homeostasis, it is reasonable to have opposite players to remove the mark on targets (eraser). Indeed, Jumonji C (JmjC) domain-containing Fe(II)/α-ketoglutarate-dependent dioxygenases are able to demethylate tri-, di-, and mono-methylated lysine of histones, named as histone lysine demethylases (KDMs) [45,46]. Coupling to diverse histone methylation marks, the JmjC protein family has a complexity with an expanded gene number and varied domain architectures [46]. Like in animals, H3K27me3 is mainly demethylated by UBIQUITOUSLY TRANSCRIBED TETRATRICOPEPTIDE X (UTX/KDM6A) and JUMONJI D3 (JMJD3/KDM6B), which both contain a JmjC domain, with extra tetratricopeptide repeat (TPR) domains in UTX [47,48]. Interestingly, JUMONJI AND AT-RICH INTERACTION DOMAIN (ARID)-CONTAINING 2 (JARID2) contains JmjN, ARID, and JmjC domains and regulates the presence of the H3K27me3 mark by recruiting PRC2 complex to chromatin, but lacks any detectable histone demethylase activity [49,50].

UTX and JMJD3 are less conserved in plants, but up to five jumonji proteins have been identified as bona fide H3K27me3 demethylases in *Arabidopsis*, including EARLY FLOWERING 6 (ELF6/JMJ11), RELATIVE OF EARLY FLOWERING 6 (REF6/JMJ12), JMJ13, JMJ30, and JMJ32 [51]. They have largely non-overlapping chromatin-targeting machineries and biological functions. Besides a JmjC domain, ELF6 also contains a zinc-finger domain that could interact with transcription factor BRASSINAZOLE-RESISTANT1 (BZR1), thus targeting to specific loci involved in the photoperiod pathway [52,53]. Similarly, its relative, REF6, is also deposited at genomic loci via zinc-finger domain and acts as a FLOWERING LOCUS C (FLC) repressor, in turn counteracting the function of ELF6 [54,55,56,57]. Conversely, JMJ13 recognizes H3K27me3 through hydrogen bonding and hydrophobic interactions and acts as a temperature- and photoperiod-dependent flowering repressor [58]. Moreover, JMJ30 and JMJ32, the JmjC domain-only group proteins, could directly bind and demethylate H3K27me3 of the *FLC* locus in vitro and in vivo, and moderate precocious flowering at elevated temperatures [59].

So far, only one H3K27me3 demethylase, OsJMJ705, has been identified in rice. Its expression could be induced by stress signals and pathogen infection. In particular, it removes H3K27me3 of defense-related genes, but also takes part in meristem development and energy-generating pathways with the aid of WUSCHEL-RELATED HOMEBOX 11 (WOX11) [60,61]. In addition, a total of 19 JmjC domain-containing proteins have been identified in maize and all are responsive to heat stress [62]; however, their molecular and biological functions remain to be explored. Hence, it will be interesting and relevant to focus on functional dissection of JmjC like H3K27me3 erasers in plants’ responses to stresses.

## 3. PRC1-Mediated H3K27me3 Reading and Gene Silencing

The canonical hierarchical model proposed that PRC2-mediated H3K27me3 does not intrinsically impact the chromatin structure but serves as a docking site for PRC1 complex [63]. PRC1 in turn catalyzes the mono-ubiquitination of histone H2A (H2Aub), thus preventing the recruitment of nucleosome remodeling factors and subsequent gene silencing (Figure 1C) [63,64,65,66,67]. In line with this notion, genome-wide chromatin binding profiles demonstrated co-occupancy of PRC1 and PRC2 at many H3K27me3 deposition loci from animals to plants [9,67,68,69,70,71,72,73]. However, evidence was also shown that some PRC1/H2Aub targeting sites are completely independent of H3K27me3 deposition, especially in plants [71,74,75,76,77]. Notwithstanding, polycomb group (PcG) proteins are tightly associated with gene repression and various biological processes.

In *Drosophila*, PRC1 contains sex combs extra (Sce), posterior sex comb (Psc), polycomb (Pc), and polyhomeotic (Ph) (Figure 1B) [78]. Of note, Sce and Psc are really interesting new gene (RING) domain-containing proteins, exhibiting E3 ubiquitin ligase activity towards histone H2A [79,80]. Pc is characterized by a chromatin organization modifier (CHROMO) domain with affinity for H3K27me3 mark [81,82]. However, the precise molecular role of essential subunit Ph has not yet been well established [83]. A burst of duplication occurs in PRC1 subunits in mammalian species, which makes the combinations more complicated (Figure 1B; Table 1). Two Sce homologs (RING1A and RING1B) have been found [84], along with six homologs of Psc in humans, termed as the B LYMPHOMA Mo-MLV INSERTION REGION 1 (BMI1) subfamily (Bmi1/Pcgf4, Mel18/Pcgf2, Nspc1/Pcgf1, Pcgf3, Pcgf5, and MBLR/Pcgf6) [85]. For Pc, at least five members are present, known as Chromobox proteins (CBX2, CBX4, CBX6, CBX7, and CBX8), conferring distinct target selectivity to the PRC1 complex [86], in contrast to three Ph homologs, PH1, PH2, and PH3 [87]. Therefore, the specificity and functions of the distinct PRC1s in mammalian species remain to be addressed.

As no significant homologs of these mammalian components were found Just as there was a lack of evidence that PRC1 is significantly homologous to mammalian species, plant PRC1 had remained elusive for a long time until the RING-finger proteins (RING1A/B and BMI1A/B/C) were characterized in *Arabidopsis* [88,89]. Double mutants of either *atring1a/b* or *atbmi1a/b* phenocopies were found in severely compromised PRC2 mutants, such as *fie*, *clf/swn* or *emf2/vrn2* mutants [90,91]. Indeed, these RING-finger proteins possess E3 ubiquitin ligase activity and are responsible for global genomic H2Aub in *Arabidopsis* [74,90,91,92,93,94]. Moreover, homologs of RING1 and BMI1 are widely present and conserved in the green lineage (Table 1) [95]. Unlike the canonical PRC1 in animals, plant PRC1 binding to H3K27me3 occurs through two distinct classes of proteins, LIKE HETEROCHROMATIN PROTEIN 1 (LHP1) and the N-terminal bromo adjacent homology (BAH) domain-containing family, which includes EARLY BOLTING IN SHORT DAYS (EBS) and its homolog SHORT LIFE (SHL), BAH DOMAIN-CONTAINING TRANSCRIPTIONAL REGULATOR 1 (BDT1), and ANTI-SILENCING 1 (ASI1)-IMMUNOPRECIPITATED PROTEIN 3 (AIPP3) [96]. Intriguingly, LHP1 was identified as a homolog of animal HP1, which contains the CHROMO domain involved in the formation of heterochromatin, but instead it functions as a Pc counterpart in plants [97,98]. In contrast, EBS and SHL are dual readers that recognize H3K27me3 and H3K4me2/3 through N-terminal BAH domain and C-terminal PHD domain, respectively [99,100,101]. Similarly, BDT1 and AIPP3 also use the BAH domain to bind to H3K27me3, facilitating the suppression of the expression of flowering genes, thus preventing flowering [102,103]. Therefore, LHP1, EBS, SHL, BDT1, and AIPP3 represent different readers of the H3K27me3 and are relevant for repression of PRC2 targets [9,97,98,99,100,101,102,103,104]. Remarkably, homologs of *Drosophila* Ph are lost in plants, instead EMF1 (phenocopying *emf2*) has been proposed to be a plant-specific PRC1 component that mediates chromatin compaction [68,105,106]. However, homologs of EMF1 appear only in dicotyledon species [95]. Phenotypes of *emf1* mutants resemble those of EMF2-PRC2 mutants [105,106,107]. In support, genome-wide binding assay revealed that PRC1 components occupy a considerable number of genes also marked with H3K27me3 [71]. Moreover, EMF1 and LHP1 are co-purified with PRC2 components and required for H3K27me3 deposition [72,101,106,108,109]. These data strongly suggest that a PRC1-like complex indeed exists in plants as well, in spite of clear divergence in function compared with animals. Notwithstanding, the mechanism underlying PRC1- and PRC2-mediated gene silencing is still far from clear in plants.

## 4. Targeting Mechanisms of H3K27me3 Deposition on Genome

The majority of PcG proteins are ubiquitously expressed in both plants and animals. However, the H3K27me3 signature is precisely and dynamically decorated at specific chromatin loci, depending on cell/tissue type, developmental stage, and environmental cues. Considered that PRC2 itself does not have DNA affinity, other essential factors are needed to determine PRC2-bound loci and spatial–temporal specificity. Accumulating evidence over recent years has revealed that diverse players, like *cis* elements, transcription factors, RNAs, and other pre-existing epigenetic modifications, are implicated in recruitment of PRC2 to target loci. However, the underlying molecular mechanisms are still not fully understood.

**Figure 2:**
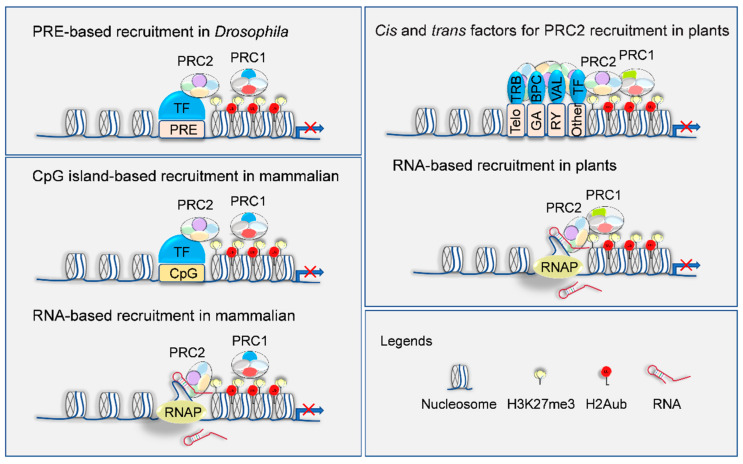
Targeting mechanisms of H3K27me3 deposition on a genome. Diagram shows PRE in the recruitment of PRC2 to target loci in *Drosophila*, CpG-island-, and RNA-based PRC2 recruitment in mammalian species, as well as *cis*- and *trans*-acting factors involved in PRC2 recruitment in plants. TF, *trans*-acting factor; RNAP, RNA polymerase. Details are explained in the text.

In *Drosophila*, polycomb response elements (PREs) could be specifically recognized by transcription factors (e.g., pleiohomeotic (Pho), GAGA factor (GAF), and Zeste) that bridge PRC2 with chromatin (Figure 2) [110]. In mammalian species, CpG islands proximal to inactive gene promoters are highly occupied by PRC2 (Figure 2) [111]. However, the mechanism directing PRC2 recruitment to CpG islands remains to be explored. In parallel, it has been observed that non-coding RNA (ncRNA) and nascent RNA directly interact with PRC2 and facilitate gene repression, probably in a SUZ12-dependent manner (Figure 2) [108,109,112]. In addition, some pre-existing epigenetic marks, such as H3K4me3, di- or tri-methylation of histone H3K36 (H3K36me2/3) and 5mC in CpG islands, counteract PRC2 recruitment in local chromatin regions [113,114,115,116]. Notwithstanding, the above-characterized mechanisms appear insufficient to account for the widespread, and often tissue-specific, H3K27me3 signature in animals. Hence, other recruiters likely also contribute to shaping H3K27 methylation patterns.

Recently, several PRE-like elements (e.g., GAGA motif, telobox motif, RY-repeat motif, and the repressive LEC2 element (RLE)) have been identified in plants (Figure 2; Table 2). For instance, GAGA motif can be recognized by BASIC PENTACYSTEINE (BPC) family members that directly interact with PcG proteins for H3K27me3 deposition [117,118,119,120,121,122], resembling the GA-repeat—GAF—module in animals [110]. Likewise, telobox motifs are bound by either TELOMERE REPEAT BINDING PROTEIN (TRB) factors or C2H2 zinc-finger family (C1-2iD subfamily) members that guide PRC2 to implement H3K27me3 deposition, which represents an evolutionarily ancient mechanism of telomeric repeats for PRC2-mediated H3K27me3 loading [120,123,124,125]. Moreover, the RY-repeat motif recruits the PRC2 complex through the intermediation of VIVIPAROUS1/ABI3-LIKE (VAL) transcription factors [126,127,128,129,130,131,132,133]. In agreement, mutants of either *trb1/2/3*, *bpc1-1bpc2bpc4bpc6,* or *val1val2* in *Arabidopsis* genetically mimic the phenotypes of those strong PRC2 mutants; however, chromatin loci occupied by these proteins jointly overlap with around 60% of H3K27me3 targets [125,129,134,135]. Therefore, additional PcG protein interactors also participate in the recruitment of PRC2 to the targeted loci, such as transcription factors, chromatin remodelers, and PRC1- and PRC2-associated factors (Table 2) [38,52,120,134,135,136,137,138,139,140,141,142,143,144,145,146,147,148,149,150,151,152,153,154,155,156,157,158,159,160,161,162,163,164,165,166,167]. Taken together, the canonical *cis*–*trans* interaction module establishes the global H3K27me3 profile and also determines the functional diversity and developmental specificity between distinct subsets of PRC2 complexes.

Although it is still controversial in regards to the molecular mechanism of the RNA-directed PRC2 recruitment in animals, several long ncRNAs have been shown to target the PRC2 complex and epigenetically silence transcription through a molecular interaction with defined loci in *Arabidopsis* (Figure 2; Table 2) [168,169,170,171,172,173]. Notably, the long ncRNAs *COLD ASSISTED INTRONIC NONCODING RNA* (*COLDAIR*), *COOLAIR,* and COLDWRAP could be induced by cold and repress transcription levels of floral repressor *FLOWERING LOCUS C* (*FLC*) via local loading of H3K27me3 [168,171,172,173]. It is likely that long ncRNAs function as a scaffold for the recruitment of chromatin-modifying factors in both plants and animals. There is a lack of any conservation of these ncRNAs; they are a general mechanism for mediating the deposition of H3K27me3 and gene silencing and by which machinery the ncRNAs recognize the chromatin are still enigmatic.

## 5. Emerging Roles of H3K27me3 in Plant Adaptation to Environmental Cues

**Figure 3:**
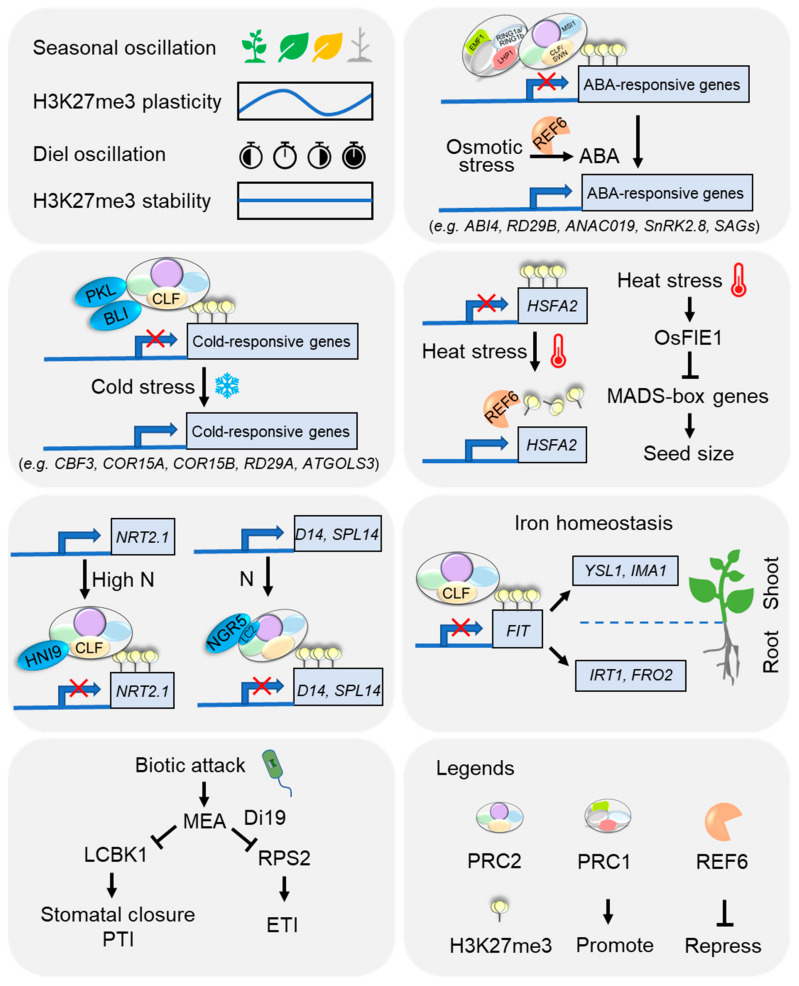
Scheme summarizing current understanding on the roles of H3K27me3 in plant adaptation to environmental cues. Acronyms and further details are explained in the text.

In parallel to their essential functions in the maintenance of cell identity and developmental phase transition in plants, PcG-protein-mediated H3K27me3 modifications are also involved in environmental stress response, either on a genome-wide scale or at specific loci [174,175,176,177,178,179,180]. Basically, changes in H3K27me3 levels are associated with transcriptional regulation of plants’ stress-responsive genes [181,182,183,184], suggesting that H3K27me3 dynamics are important in the regulation of plant adaption to environmental cues (Figure 3).

### 5.1. Seasonal and Diel Oscillations

Seasonal and diel oscillations are two major environmental inputs into plant growth and development. Genome-wide chromatin immunoprecipitation sequencing (ChIP-seq) studies have demonstrated that H3K27me3 exhibits seasonal plasticity and diel stability, suggesting that H3K27me3 might act as a monitor to sense the fluctuating environmental stresses, in turn adjusting gene expression and regulation [185,186,187].

A prominent case is vernalization, a naturally occurring phenomenon that promotes flowering by a certain prolonged exposure to cold/low temperatures in winter. Specifically, PcG-protein-mediated histone modifications and epigenetic silencing of the main floral inhibitor *FLC* are the central hub in the control of vernalization, which is conserved in several plant species [188]. Studies regarding epigenetic regulation of the *FLC* locus have been thoroughly discussed in several reviews [175,189,190,191], and will not be discussed here.

### 5.2. Extreme Temperature

Temperature is a major environmental factor that greatly influences plant growth and development. Cold stress could increase chromatin accessibility by coordinating the bivalent H3K4me3 and H3K27me3 modifications, thereby activating cold-responsive gene expression in potatoes (*Solanum tuberosum* L.) [192]. In *Arabidopsis*, a CLF-interacting protein BLISTER (BLI) promotes the resistance to cold stress [181]. Moreover, the chromatin remodeler PICKLE (PKL) is required for proper chilling and freezing tolerance, through a CLF-related H3K27me3 pathway [193,194]. Indeed, PKL regulates the expression of many cold-responsive genes, including *C-REPEAT BINDING FACTOR 3* (*CBF3*), *RESPONSIVE TO DESICCATION 29A* (*RD29A*), *COLD-RESPONSIVE 15A* (*COR15A*), and *COR15B* [194]. In line with this, cold exposure triggers a significant decrease in H3K27me3 levels of cold-responsive genes *COR15A* and *GALACTINOL SYNTHASE 3* (*ATGOLS3*), and intriguingly the H3K27me3 amount could be stably maintained to normal growth conditions; hence, they likely serve as epigenetic memory markers for recent transcriptional activity in *Arabidopsis* [195].

Similarly, heat stress also seriously impairs the growth and production of plants. Conversely to cold, heat can erase the epigenetic marks established during vernalization in *Arabidopsis* [196]. Mechanically, the heat-induced HEAT SHOCK TRANSCRIPTION FACTOR A2 (HSFA2) directly activates the expression of H3K27me3 demethylase REF6 to reduce the H3K27me3 level at *HSFA2* loci, which further enhances the expression *HSFA2*, thereby forming a REF6-HSFA2 regulatory loop orchestrating transgenerational thermomemory in *Arabidopsis* [184]. Whilst PRC2-mediated H3K27me3 modification controls the early endosperm development, it also accounts for the reduced seed size and yield under heat stress in both *Arabidopsis* and cereals [197]. Importantly, heat stress could misregulate *OsFIE1* expression and alter the duration of syncytial stage endosperm development, whereas overexpression of *OsFIE1* leads to reduced seed size as a result of precocious cellularization [198,199].

### 5.3. Nutrients

Nutrient availability from the diverse and variable environment is vital for plant growth and crop performance. Of which, nitrogen (N), one of the most important major mineral nutrients, is perceived and transported mainly by nitrate transporters (NRTs) [200]. *NRT2.1*, encoding a main component of the root high-affinity transport system for NO_3_^−^, is repressed under high N supply in a HIGH NITROGEN INSENSITIVE 9 (HNI9)-dependent manner, with increased levels of H3K27me3 at the *NRT2.1* site in *Arabidopsis* [201]. In support of this, mutation of *CLF* leads to a loss of H3K27me3 at the *NRT2.1* loci and enhanced N absorption [202]. Moreover, genome-wide analysis revealed that CLF-related H3K27me3 targets were significantly enriched in metabolic processes, in response to diverse stimuli, in nitrate transport and assimilation, as well as in mineral nutrition and secondary metabolism [202]. In addition, NITROGENMEDIATED TILLER GROWTH RESPONSE 5 (NGR5), an APETALA2-type transcription factor, is able to interact with a component of PRC2 and alter the genome-wide H3K27me3 levels in response to changes in nitrogen availability in rice [165].

Iron is an essential micronutrient but overdose can lead to toxicity. PRC2-mediated H3K27me3 modulates iron homeostasis in *Arabidopsis* as well. Notably, FER-LIKE IRON DEFICIENCY-INDUCED TRANSCRIPTION FACTOR (FIT), directly targeted by H3K27me3, is a master regulator of iron deficiency response [203]. Moreover, genome-wide analysis showed that CLF regulates the expression of FIT-dependent iron acquisition genes, like IRON-*REGULATED TRANSPORTER 1* (*IRT1*) and *FERRIC REDUCTASE OXIDASE 2* (*FRO2*) in roots, and also iron homeostasis genes (e.g., *YELLOW STRIPE-LIKE 1* (*YSL1*), *IRON MAN 1* (*IMA1*)) in shoots, thereby modulating iron translocation from roots to shoots; Consistently, *clf* mutants have been found to be more resistant to low-iron conditions than the wild type [203,204].

### 5.4. Osmotic Stress

Osmotic stresses, such as drought and salinity, are major factors restricting plant growth and development, survival and distribution, and are associated with the H3K27me3 mark [2]. Surprisingly, it has been reported that H3K27me3 is not responsible for the transcription reprogramming of stress-responsive genes under dehydration stress [177,205,206,207]. Notwithstanding, impairments of either *MSI1* or *LHP1* increase drought tolerance in *Arabidopsis*, probably through the abscisic acid (ABA) signaling pathway [182,208]. In contrast to drought, salt stress can greatly alter H3K27me3 patterns in different plant species [206,209,210,211]. In line with this, reduction of *MSI1* expression or *EMF1* activity can enhance salt tolerance, through upregulation of H3K27me3 targets, like ABA receptor genes and ABA-responsive genes [212,213].

As a key growth regulator, ABA plays pivotal roles in plant drought- and salt-stress responses [214]. Intriguingly, H3K27me3 demethylase REF6 has been shown to induce ABA biosynthesis in *Arabidopsis* seeds [215]. In line with removal of H3K27me3, a number of ABA-responsive genes (e.g., *ABA-INSENSITIVE 4* (*ABI4*), *RESPONSIVE TO DESSICATION 29B* (*RD29B*), *NAC DOMAIN CONTAINING PROTEIN 19* (*ANAC019*), *ANAC055*, *SNF1-RELATED PROTEIN KINASE 2.8* (*SnRK2.8*)) and ABA-induced senescence-associated genes (SAGs) are induced and involved in stress responses [181,182,216,217,218]. By contrast, mutants of *clf-50 swn-1*, *msi1-cs, atring1a atring1b,* and *lhp1* are all hypersensitive to ABA, which demonstrates the crucial roles of PcG proteins in attenuating ABA signaling [184,211,219,220].

### 5.5. Biotic Stress

In addition to abiotic stress, plants are also subjected to many biotic attacks from bacteria, fungi, oomycetes, viruses, and insects. Therefore, plants evolve a two-layered innate immune system consisting of the pattern-triggered immunity (PTI) and the effector-triggered immunity (ETI). PTI and ETI result in many overlapping downstream outputs, such as reactive oxygen species (ROS) burst, programmed cell death (PCD), transcriptional reprograming, and phytohormone signaling (e.g., salicylic acid (SA) and jasmonate acid (JA))[221]. Besides, genome-wide H3K27me3 atlas was observed in plant responses to infection by pathogens [219,220,222,223,224]. The expression of *MEA* could be induced by either pathogen inoculation or exogenous application of JA or SA. Indeed, MEA can suppress both PTI and ETI in *Arabidopsis* [156,183]. Specifically, MEA is able to interact with LONG-CHAIN BASE KINASE1 (LCBK1) and impair its function, which in turn results in a loss of pathogen-induced stomatal closure and PTI; meanwhile, MEA could be recruited by DROUGHT-INDUCED 19 (Di19) to implement H3K27me3 modification on the immune receptor *RESISTANCE TO P. SYRINGAE2* (*RPS2*) loci, thereby repressing its expression and attenuating AvrRpt2 effector-mediated ETI [156,183]. Consistently, *MEA*-overexpressing transgenic plants are susceptible to fungal pathogens, bacterial pathogens, and Pst-AvrRpt2, whereas *mea-6* mutant plants are more resistant to bacterial pathogens [156,183]. Moreover, loss of *SWN* caused a significantly increased hypersensitive response (HR) during the time course of AvrRpt2 induction, revealing a role of SWN in attenuating PCD [224]. In rice, the H3K27me3 demethylase, *OsJMJ705*, could be induced by stress signals and pathogen infection. Overexpression of *OsJMJ705* derepresses H3K27me3-marked biotic stress-responsive genes and enhances rice resistance to the bacterial blight disease pathogen *Xanthomonas oryzae pathovar oryzae*, in contrast to reduced resistance of its mutant [60]. Altogether, these observations imply that PRC2-mediated H3K27me3 modification plays negative roles in plant pathogen defense, probably through blocking the phase transition from growth to senescence.

## 6. Conclusions and Perspectives

H3K27me3, a hallmark of gene silencing, plays prominent roles in cell identity control and developmental phase transition, both in animals and in plants. PRC2 complex, which is evolutionarily conserved across different lineages, catalyzes H3K27me3 modification, which is sequentially recognized by the PRC1 complex, thereafter raising the H2Aub mark and chromatin compaction. In contrast to PRC2, components and machinery of PRC1 appear less conserved between two aspects of animals and plants. Firstly, they utilize different kinds of readers to decode H3K27me3 mark; secondly, unlike the animal hierarchical model, plant PRC1 and PRC2 likely cooperate to exert H3K27me3 deposition and gene repression. Regarding erasing histone lysine methylation, Jumonji C domain proteins have context-dependent substrate specificity toward various histone lysine sites. Particularly, members of the H3K27me3-specific demethylase family identified in plants are relatively limited. Hence, great efforts should be made to address this question. Moreover, writers, erasers, and readers of H3K27me3 need to be functionally characterized in more plant species other than *Arabidopsis*, like monocot crops. Further investigations in this area promise to unravel conservation and functional diversification of H3K27me3 machinery during evolution of eukaryotic organisms.

In both plants and animals, *cis* and *trans* determinants are required for PRC2 recruitment and H3K27me3 deposition. In spite of the limited conservation, some common factors, like GAGA motif and telomeric repeats, do exist across kingdoms. This raises the question when and how eukaryotic cells obtain the ancestral *cis*-localized DNA sequence motif pathway for H3K27me3 loading. In parallel, *trans*-acting factors are also promising mediators for recruiting PRC2 through interaction with components of PRC2 and/or PRC1. Importantly, transcription factors play key roles in determining the specificity of H3K27me3 dynamics in different stages of growth and development, as well as responses to various environmental cues. Notwithstanding, we are still far from reaching a full understanding of these open questions. Therefore, research in this direction is expected to identify new regulators for H3K27me3-mediated gene repression, especially in stress responses. In addition, PRC2 can also methylate non-histone proteins. It will be fascinating to discriminate whether some of the interacting proteins are directly methylated by PRC2 independent of H3K27me3.

In genetic regulatory networks, epigenetic mechanisms are of great significance in fine-tuning gene expression in plants’ responses to environmental cues. However, reports about the roles of the H3K27me3 dynamics in plant environmental adaption are quite limited, except the vernalization pathway in *Arabidopsis*. Undoubtedly, recent advances in high-throughput sequencing technologies using small amounts of chromatin DNA, even at the single-cell level, will open new avenues for expanding our understanding of reprogramming of H3K27me3 in plant stress responses. As polycomb-mediated H3K27me3 regulation mostly plays a negative role in stress responses, it highlights a possible role of H3K27me3 in balancing plant growth and adaptation to stress. Nevertheless, more evidence is needed to ascertain this hypothesis. The in-depth knowledge on H3K27me3 regulation in plants promises to provide new candidates and methods for enhancing crop productivity under stressful environments.

## Figures and Tables

**Table 1:** PRC1 and PRC2 core complex components in animals and plants.

Drosophila	Characteristic Domains	Human	Arabidopsis	Rice	Maize
**Polycomb Repressive Complex 2 (PRC2)**					
E(z)	SET, CXC and SANT	EZH1, EZH2	CLF, SWN, MEA	OsCLF, OsiEZ1	ZmMEZ1, ZmMEZ2, ZmMEZ3
Esc	WD40	EED	FIE	OsFIE1, OsFIE2	ZmFIE1, ZmFIE2
Su(z)12	Zinc-finger and VEFS	SUZ12	FIS2, VRN2, EMF2	OsEMF2a, OsEMF2b	ZmEMF2a, ZmEMF2b
p55	WD40	RBBP4, RBBP7	MSI1–5	OsMSI1, OsMSI3, OsMSI4	ZmMSI1a/b/c/d; ZmMSI3; ZmMSI4a/b
**Polycomb Repressive Complex 1 (PRC1)**					
Sce	RING	RING1A, RING1B	AtRING1A/B	OsRING1A/B	ZmRING1A/B/C/D
Psc	RING	PCGF1–6	AtBMI1A/B/C	OsBMI1A/B/C	ZmBMI1A/B/C/D/E/F
Pc	CHROMO	CBX2/4/6/7/8	–	–	–
Ph	SAM and Zinc-finger	PH1–3	–	–	–
	CHROMO		LHP1	OsLHP1	ZmLHP1
			EMF1	–	–

–, No homologues found; further acronyms and details are explained in the text.

**Table 2:** *Cis*- and *trans*-acting factors involved in PRC2 recruitment in plants.

*Trans*-Acting Factor	Interacting PcG Protein	*Cis*-Acting Factor	Targeted Loci	Biological Function	Reference
**Transcription factors**					
BPC1/2/4/6	SWN	N	*ABI4*	Lateral root development	[119]
BPC class I subfamily	FIE	GAGA motif	N	N	[118,120]
BPC6	LHP1, VRN2	GAGA motif	Homeotic genes	Vegetative growth and flowering	[117]
BPC1/2/3	FIE, MEA, FIS2, MSI1	GAGA motif	*FUSCA3*	Seed development	[122]
N	N	W-box, RY motif	*FUSCA3*	Seed development	[131]
BBR/BPC	N	GAGA motif	N	Brassinosteroid signaling	[121]
TRB1/2/3	CLF, SWN	Telobox	N	Vegetative growth and flowering	[123,124]
C2H2 ZnF family	FIE	Telobox	N	N	[120]
AP2 subfamily	FIE	PRE-like	N	N	[120]
VAL1/2	CLF, SWN	RY repeat	N	Somatic embryonic calli	[132]
VAL1/2	MSI1, LHP1	N	*FT*, *FLC*	Flowering	[126,127]
VAL1/2	CLF, MSI1, LHP1	RY repeat	*DOG1*, *AGL15*	Seed germination and vegetative growth	[128,129]
N	N	RLE	*LEC2*	Embryo development	[164]
AS1/2	CLF, FIE, EMF2, LHP1	N	*BP*, *KNAT2*	Leaf differentiation	[140,163]
PWO1	CLF, SWN, MEA	N	N	Vegetative growth and flowering	[157]
PDP1/2/3	MSI5	N	*FLC*	Flowering	[138]
JAZ1/4/8/10/3/6/9	EMF2, LHP	N	*DYT1*, *AMS*, *MS1*, *JAZ1*	Jasmonate signaling	[151]
PPD1/2	LHP1	N	D3-type cyclins and *HMGA*	Lateral organ growth	[166]
NGR5	LC2	GCCGCC motif	*D14*, *SPL14*	Tillering from nitrogen regulation	[165]
AG	LHP1	Chromatin loop	*WUS*	Meristem maintenance and determinacy	[142]
KNU	FIE	N	*WUS*	Floral meristems determinacy	[150]
Di19	MEA	N	*RPS2*	Pathogen defense	[156]
RBR1	FIE, MSI	N	*MET1*	Female gametogenesis	[139,147,159]
OsRBR1/2	OsMSI1	N	N	Floral development	[160]
ESD7	CLF, EMF2, MSI1	N	*FT*, *SOC1*	Vegetative growth and flowering	[134]
**Chromatin remodelers**					
BLI	CLF	N	Homeotic genes, *FLC*, *FT*	Vegetative growth and flowering	[144]
ICU11	CLF, SWN, EMF2, MSI1, FIE	N	*FLC*	Flowering	[135]
ALP2	MSI1	N	N	Vegetative growth and flowering	[146]
EOL1	CLF, SWN, LHP1	N	N	H3K27me3 inheritance	[143]
OsCTF4	OsCLF, OsLHP1, OsSWN	N	*KRP1*, *KRP5*	Cell cycle and vegetative growth	[161]
ATX1	CLF	N	*AG*	Vegetative development	[136]
TAF13	MEA, SWN	N	*PHE1*, *FUS3* and *AtFH5*	Seed development	[162]
SVP	LHP1	N	*SEP3*	Floral patterning	[158]
ATRX	LHP1	N	*FLC*	Flowering	[153]
**PcG-associated proteins**					
VIN3, VEL1, VRN5	VRN2	N	*FLC*	Flowering	[38]
OsVIL3	OsVIL2	N	*OsLF*	Rice flowering	[152]
OsVIL2	OsEMF2b	N	*OsLFL1*, *OsTB1*	Rice flowering	[141,154,155]
DFO1	OsMSI1, OsiEZ1	N	*OsMADS58*	Floral organ identity	[145]
**ncRNAs**					
N	CLF	AG-incRNA4	*AG*	Tissue specification	[169]
N	LHP1	APOLO	*PID*	Auxin signaling	[170]
FCA	CLF	COOLAIR	*FLC*	Flowering	[171]
N	CLF	COLDAIR	*FLC*	Flowering	[168,172]
N	CLF	COLDWRAP	*FLC*	Flowering	[173]

N, not detected or not applicable. References listed should be consulted for further details.
